# Patient Use of Physicians’ First (Given) Name in Direct Patient Electronic Messaging

**DOI:** 10.1001/jamanetworkopen.2022.34880

**Published:** 2022-10-05

**Authors:** Jamison A. Harvey, Richard J. Butterfield, Shari A. Ochoa, Yul W. Yang

**Affiliations:** 1 Department of Dermatology, Mayo Clinic, Scottsdale, Arizona; 2 Department of Quantitative Health Sciences, Mayo Clinic, Scottsdale, Arizona

## Abstract

This cohort study examines factors that may contribute to whether patients address physicians differently through electronic messaging

## Introduction

Physicians are typically formally addressed as “Doctor” by patients, acknowledging the physician-patient relationship, signifying respect for physicians, and following established social norms. In a previous survey of 333 physicians, almost three-quarters of respondents reported being called by their first (given) name, with annoyance reported in 61%.^1^ A recent study revealed that having “DOCTOR” identification badge labels were associated with female physicians and physicians underrepresented in medicine experiencing substantially fewer episodes of bias from misidentification.^2^ Here, we aim to determine factors that are associated with whether patients addressed physicians differently through electronic messaging.

## Methods

After approval by the Mayo Clinic institutional review board with waiver of participant consent because of exempt status, a retrospective review was conducted of patient messaging to physicians through the Mayo Clinic electronic medical record from October 1, 2018, to September 30, 2021. This cohort study followed the Strengthening the Reporting of Observational Studies in Epidemiology (STROBE) reporting guideline. Messages were evaluated using a natural language processing algorithm to identify the greeting and/or closing salutation used by patients and to classify these greetings based on formality. We defined a formal greeting as “Dr. (or Doctor) Last-name” or “Dr. (or Doctor) First-name Last-name.” Available demographics of patients (age and gender) and physicians (age, gender, degree, training level, and specialty) were determined.

Use of formal greeting was compared between women and men physicians using χ^2^ test. Multilevel logistic regression analysis of first name greeting with patients nested within physicians was performed for available patient and physician demographic variables univariately and in a multivariable framework. Two-tailed *P* < .05 was considered statistically significant. All analyses were performed in SAS version 9.4 (SAS Institute).

## Results

A total of 90 830 messages from 34 829 patients were identified. Of those, 29 498 messages (32.5%) from 14 958 patients included the physician’s name in the greeting or closing salutation (Table 1).

**Table 1.** Patient and Physician Demographics

Characteristic	Participants, No. (%)
**Patients**	
Total No.	14 958
Patient age, y	
Mean (SD)	55.0 (19.2)
Median (IQR)	59 (44-69)
(Range)	(0.0-104.0)
Patient gender, No. (%)	
Men	6383 (42.7)
Women	8575 (57.3)
**Physician demographics**	
Total No.	1092
Physician age, y	
Mean (SD)	39.3 (11.3)
Median (IQR)	35 (30, 46)
(Range)	(24.0-78.0)
Physician gender, No. (%)	
Men	524 (48.0)
Women	568 (52.0)
Degree, No. (%)	
MD	983 (90.0)
DO	72 (6.6)
MBBS	37 (3.4)
Training level, No. (%)	
Physician	764 (70.0)
Resident or fellow	328 (30.0)
Practice setting, No. (%)	
Primary care	506 (46.3)
Specialty	586 (53.7)

Abbreviations: DO, doctor of osteopathic medicine; MBBS, bachelor of medicine, bachelor of surgery; MD, doctor of medicine.

There were messages to 1092 physicians (568 [52.0%] female) (Table 1). Women physicians had more than twice the odds as men to be called by their first name after adjusting for patient gender; physician age, degree, level, and specialty; and for messages sent on behalf of the patient (odds ratio [OR], 2.15; 95% CI, 1.68-2.74; *P* < .001) (Table 2). Physicians holding a doctor of osteopathic medicine (DO) degree had nearly twice the odds (OR, 1.86; 95% CI, 1.20-2.88; *P* = .006) and primary care physicians had approximately 50% greater odds to be called by their first name (OR, 1.48; 1.16-1.89; *P* = .002). Women patients had approximately 40% lower odds to address their physicians by first name (OR, 0.57; 95% CI, 0.50-0.65; *P* < .001). There was no difference based on patient or physician age or whether the physician was in training (resident or fellow) (Table 2).

**Table 2.** Univariate and Multivariable Logistic Regression Mixed Model Analysis of First Name–Only Physician Greeting

Variable	Level	Univariate		Multivariable	
		OR (95% CI)	*P* value	OR (95% CI)	*P* value
Patient age	1-y increase	1.00 (1.00-1.00)	.31	NA	NA
Patient gender	Women vs men	0.60 (0.53-0.68)	<.001	0.57 (0.50-0.65)	<.001
Physician age	1-y increase	0.99 (0.98-1.00)	.009	0.99 (0.98-1.00)	.22
Physician gender	Women vs men	2.10 (1.65-2.67)	<.001	2.15 (1.68-2.74)	<.001
Physician degree	DO vs MD	2.00 (1.29-3.12)	.002	1.86 (1.20-2.88)	.006
	MBBS vs MD	0.73 (0.30-1.80)	.49	0.90 (0.36-2.23)	.82
Physician level	Resident or fellow vs attending physician	1.22 (0.93-1.61)	.15	1.03 (0.75-1.43)	.84
Specialty	Specialty vs primary care	0.60 (0.47-0.76)	<.001	0.68 (0.53-0.86)	.002

Abbreviations: DO, doctor of osteopathic medicine; MBBS, bachelor of medicine, bachelor of surgery; MD, doctor of medicine; NA, not applicable.

## Discussion

To our knowledge, this is the first study to objectively identify patterns of addressing physicians through electronic messaging and may reveal potential bias. We found that women physicians, DO physicians, and primary care physicians were addressed by their first name more frequently.

There are several limitations to our study. We were unable to control if physicians prefer to be addressed informally and for potential cultural, racial, or ethnic nuances in greeting structure. We relied on natural language processing to isolate physician greetings, which may not identify all greetings appropriately. Because this was performed through one health system, the results may not be generalizable.

The pattern of addressing physicians with different titles based on gender, degree, and specialty may be forms of bias.^3,4^ Similar studies at grand rounds or academic meetings have found women were less likely to be addressed by a full professional title when introduced by men.^5,6^

Whether being informally addressed by other medical professionals or patients, untitling (not using a person’s proper title) may have a negative impact on physicians, demonstrate lack of respect, and can lead to reduction in formality of the physician-patient relationship or workplace. Institutional leadership and individual efforts should focus on a supportive culture, with particular attention spent to address potential unconscious biases as revealed here. Such efforts could include formal guidelines, practice changes, direct patient education, and further research to explore other areas of unconscious bias.

## References

[1] Farber NJ, Novack DH, Silverstein J, Davis EB, Weiner J, Boyer EG. Physicians’ experiences with patients who transgress boundaries. J Gen Intern Med. 2000;15(11):770-775. doi:10.1046/j.1525-1497.2000.90734.x 11119168 PMC1495619

[2] Olson EM, Dines VA, Ryan SM, . Physician identification badges: a multispecialty quality improvement study to address professional misidentification and bias. Mayo Clin Proc. 2022;97(4):658-667. doi:10.1016/j.mayocp.2022.01.007 35379420

[3] Brown J, Drury L, Raub K, . Workplace harassment and discrimination in gynecology: results of the AAGL member survey. J Minim Invasive Gynecol. 2019;26(5):838-846. doi:10.1016/j.jmig.2019.03.004 30878643

[4] Chilakala A, Camacho-Rivera M, Frye V. Experiences of race- and gender-based discrimination among Black female physicians. J Natl Med Assoc. 2022;114(1):104-113. doi:10.1016/j.jnma.2021.12.008 35058066

[5] Files JA, Mayer AP, Ko MG, . Speaker introductions at internal medicine grand rounds: forms of address reveal gender bias. J Womens Health (Larchmt). 2017;26(5):413-419. doi:10.1089/jwh.2016.6044 28437214

[6] Duma N, Durani U, Woods CB, . Evaluating unconscious bias: speaker introductions at an international oncology conference. J Clin Oncol. 2019;37(36):3538-3545. doi:10.1200/JCO.19.01608 31603705

